# The clinical significance and anti-tumor role of PRKG1 in bladder cancer

**DOI:** 10.3389/fimmu.2024.1442555

**Published:** 2024-07-30

**Authors:** Lu Jin, Ting Chen, Huan Sun, Guangcheng Dai, Qiu Yao, Feng Yuan, Xiaolong Liu, Boxin Xue

**Affiliations:** ^1^ Department of Urology, the Second Affiliated Hospital of Soochow University, Suzhou, China; ^2^ Department of Pathology, Children’s Hospital of Soochow University, Suzhou, China; ^3^ Department of Pathology, the Second Affiliated Hospital of Soochow University, Suzhou, China

**Keywords:** PRKG1, bladder cancer, immunotherapy, BCG, prognosis

## Abstract

**Introduction:**

cGMP-dependent protein kinase 1 (PRKG1) has shown to be associated with some tumorigenesis, while the role of PRKG1 in bladder cancer is unclear.

**Methods:**

To investigate the biological and clinical significance of PRKG1 in bladder cancer, we detected the expression of PRKG1 and explored the function of PRKG1 in bladder cancer cells. The PRKG1 transcripts data was downloaded from The Cancer Genome Atlas (TCGA) database, and immunohistochemistry staining was conducted on formalin-fixed paraffin-embedded (FFPE) sample tissues. Relationship between clinical characteristics of patients and expression of PRKG1 was analyzed in FFPE samples, TCGA database, and GSE19423 dataset. PRKG1 was over-expressed, and cell proliferation, migration, invasion, apoptosis, and spheroidizing ability were then detected. Chemosensitivity to cisplatin was detected with cell viability, and half-maximal drug inhibitory concentration (IC50) was calculated. In addition, the relation between PRKG1 expression and the infiltration level of tumor immune cells in tumor microenvironment were analyzed.

**Results:**

The results showed expression of PRKG1 was lower in bladder cancer, compared with normal tissues both at protein and transcript levels. Lower PRKG1 expression was related to higher tumor grade, T stage, and muscle invasion, also predicted worse overall survival and recurrence free survival in patients treated with Bacillus Calmette–Guerin (BCG) intravesical immunotherapy. Analysis of tumor immune cells infiltration showed lower PRKG1 was associated with non-inflamed tumor microenvironment.

**Conclusion:**

The present study firstly identified the anti-tumor role and tumor immune regulatory role of PRKG1, also found loss of PRKG1 could be used as a prognosis factor. The present study provided a potential biomarker and therapy target to bladder cancer.

## Introduction

Bladder cancer (BCa) is the second most common urological tumor worldwide, accounting for 613,791 new incidence and 220,349 mortalities in 2022 (1). About 75% new cases were diagnosed with non-muscle-invasive bladder cancer (NMIBC), and the remaining 25% patients were cases of muscle-invasive bladder cancer (MIBC) (2). NMIBC has a better overall survival rate, while the recurrent rate is high (50% to 70%), and 10%–20% of cases progress to MIBC (2). For MIBC patients, radical cystectomy (RC) combined with cisplatin-based neoadjuvant therapy is advised (2). In MIBC patients who do not accept RC or for whom RC is not an option, it is advised that they receive multimodal bladder-preserving treatments (TURBT, cisplatin-based chemotherapy, and others). Transurethral resection of bladder tumor (TURBT) is the main therapy of NMIBC, and patients always receive intravesical instillations after TURBT to prevent recurrence and progression. Maintenance intravesical chemotherapy or Bacillus Calmette–Guerin (BCG) immunotherapy is the recommended option. The drugs used in intravesical chemotherapy include epirubicin, gemcitabine, pirarubicin, hydroxycamptothecin, mitomycin-C, etc. (3). For patients with intermediate- to high-risk tumors, intravesical BCG immunotherapy is recommended, while for patients with intermediate-risk tumors, intravesical chemotherapy is recommended (3). Intravesical BCG immunotherapy has shown superior efficacy in reducing tumor recurrence and progression compared to adjuvant intravesical chemotherapy (4). Unfortunately, about 40% patients do not respond to BCG treatment (5). Currently, cisplatin-based chemotherapy and immunotherapy are common options for BCa therapy, which is not beneficial to all patients. Autophagy, EMT, tumor stemness, aberrant tumor microenvironment (TME), and some other cellular progressions have been thought to be related to therapy resistance (6)—for example, SIRT1 induces cisplatin resistance in bladder cancer T24 cells through Beclin1-deacetylation-mediated autophagy activation (7). As a consequence, identifying reliable biomarkers based on the mechanism to estimate prognosis and drug sensitivity and guide individual-based therapy is essential. The cGMP-dependent protein kinase (PKG), a serine/threonine-specific protein kinase, is recognized as a critical regulator in the modulation of neuronal functions, inhibition of platelet activation, and maintenance of bone homeostasis (8). Additionally, PKG was also reported to be with antiproliferative/apoptotic effect in some cells, including cardiomyocytes, neutrophils, and mesangial cells (9, 10). Type 1 PKG (PRKG1) was identified as one of the PKG isoforms, which arose from alternative splicing and was widely distributed in eukaryotes. Some researchers focused on the role of PKG in some cancers, but only a few studies detected the clinical significance of PRKG1. Hou et al. found that tumors might have a decreased expression of PRKG1 compared with normal tissues (11). In epithelial ovarian cancer, increased endogenous PRKG1 activity attenuates EGF-induced proliferation and migration via the MAPK/ERK pathway (12). However, the expression panels of PRKG1 in urothelial bladder cancer remain unclear. In the present study, we detected the expression of PRKG1 in bladder cancer tissues and explored the role of PRKG1 in bladder cancer progression and therapy resistance.

## Materials and methods

### Patients and tissue samples

Following the approval from the Ethics Committee of the Second Affiliated Hospital of Soochow University, samples derived from patients diagnosed with primary urothelial bladder cancer were collected. A total of 115 TURBT samples were collected from January 2018 to December 2019. Moreover, 59 RC samples and 19 paired samples were collected from January 2017 to December 2020. The original diagnosis was confirmed by a urologic pathologist according to the World Health Organization/International Society of Urological Pathology (WHO/ISUP) consensus classification of urothelial neoplasms of the urinary bladder. After obtaining ethics approval from the institutional review board, we retrospectively reviewed the detailed clinical information of these patients. Additionally, the samples were constructed by paraffin-embedded, formalin-fixed bladder cancer specimens. The clinical characteristics of the patients can be found in **Table 1**.

**Table 1:** The relationship between PRKG1 and clinicopathologic characteristics in patients with bladder cancer from FFPE cohort.

Parameter	N	Low (70)	High (104)	*P*
Age				0.44
<65	74	27 (15.52%)	47 (27.01%)	
≥65	100	43 (24.71%)	57 (32.76%)	
Gender				0.28
Female	25	13 (7.47%)	12 (6.90%)	
Male	149	57 (32.76%)	92 (52.87%)	
Grade				< 0.001
Low	93	20 (11.49%)	73 (41.95%)	
High	81	50 (28.74%)	31 (17.82%)	
T stage				< 0.001
Ta/1	91	19 (10.92%)	72 (41.38%)	
T2	68	42 (24.14%)	26 (14.94%)	
T3/4	15	9 (5.17%)	6 (3.45%)	
Muscle Invasion				< 0.001
NMIBC	91	19 (10.92%)	72 (41.38%)	
MIBC	83	51 (29.31%)	32 (18.39%)	

### Western blot and immunohistochemistry

Immunohistochemistry staining was performed on urothelial bladder cancer tissues at the Second Affiliated Hospital of Soochow University. The tissue blocks were cut at sections and were deparaffinized in xylene and ethanol after baking for 60 min. The deparaffinized sections were then brought in 0.01 mol/L sodium citrate buffer and boiled for 30 min. The sections were further incubated with 3% H_2_O_2_ for 10 min, blocked with 10% goat serum for 15 min, and incubated with primary antibodies (4°C, overnight). After washing with PBS three times, the sections were incubated with secondary antibody for 20 min. Then, these sections were stained using DAB Substrate kit and counterstained with hematoxylin. All stains were manually evaluated by two experienced pathologists who were blinded to sample identification independently. The German Immunoreactive Score was accessed by multiplying the staining intensity (0, negative; 1, weak; 2, moderate; 3, strong) by percentage of immunoreactive cells (0% = 0, 1%–10% = 1, 11%–50% = 2, 51%–80% = 3, and 81%–100% = 4). The interpretation score was defined as follows: 0–1 (0, negative), 2–4 (1+, weakly positive), 6–8 (2+, moderately positive), and 9–12 (3+, strongly positive). The cells were collected, and protein was extracted with RIPA lysis buffer (Beyotime, Beijing, China). Western blotting was performed as described previously (7). A total of 30 mg protein was used for blotting. β-Actin was used for the loading control, and antibodies were obtained from Proteintech China (Hubei, China).

### Database and preprocessing

BLCA RNA sequencing data (fragments per million reads mapped, FPKM value) and somatic mutation data in The Cancer Genome Atlas (TCGA) database were downloaded from the UCSC Xena platform (https://xena.ucsc.edu/) (13). TCGA–BLCA RNA-seq data were shown as FPKM or log_2_(*x* + 0.001) transformed, and VarScan2 was used to analyze and calculate tumor mutation burden (TMB). The microsatellite instability (MSI) score and DNA stemness score (DNAss) were obtained from previous studies (14, 15). BCa with BCG intravesical immunotherapy cohort (GSE19423) was downloaded from the Gene Expression Omnibus database. The expression of PRKG1 and the transcripts was downloaded from Genotype-Tissue Expression (GTEx).

### Evaluation of TME in BLCA

The infiltration level of tumor-infiltrating immune cells in TME was calculated with TIMER algorithm. The ESTIMATE algorithm was used to estimate the stromal and immune scores for each sample. The expression of immunomodulators was obtained from the TCGA–BLCA datasets.

### Cell culture and transfection

The cell lines (5637 and T24) used in the present study were maintained in DMEM supplemented with 10% FBS and 1% penicillin streptomycin at the temperature of 37°C, with 5% CO_2_. The cells were cultured for about 24 h and then transfected with lentivirus particles (2 * 10^6^ TU), including PRKG1 sequence or NC. At 48 h later, the medium was replaced with new complete medium. The PRKG1 lentivirus particles and control lentivirus were purchased from Genechem (Shanghai, China). qPCR and Western blot were performed to verify the over-expression of PRKG1 mRNA and protein after treatment with PRKG1 lentivirus particles.

### Wound healing assay

About 50,000 cells were seeded into a six-well plate, and at 24 h later, an artificial wound was scratched on the well surface with a 200-μL pipette tip. Then, the cells were cultured in complete medium. Images of the scratches were obtained with a digital camera system at 0, 12, and 24 h.

### Cell invasion assay

Transwell invasion assay was performed with Transwell chambers (6.5 mm; Corning, USA) in a 24-well plate. Extracellular matrix (70 μL Matrigel, 1 mg/mL, BD Biosciences, USA) was added on top of the porous membrane which only permits chemotaxis of cells which possess invasive properties. The lower chamber was filled with 600 μL of culture medium (supplemented with 30% FBS), and 2 × 10^5^ cells were suspended with 100 μL of medium (medium with 1% FCS) and added into the Transwell insert. At 24 h later, cells that invaded the lower surface of the Transwell chambers were fixed and stained with crystal violet. Using a microscope, multiple images of the Transwell membrane were taken, and the number of invaded cells could be used to indicate invasive ability.

### Tumor sphere formation assay

Cells were seeded in an ultralow-attachment six-well plate at a density of about 1,000 cells per well, with DMEM/F12 medium (GIBCO), B27 (10 µL/mL, Life Technology), human EGF (20ng/mL, PeproTech), and human bFGF (10 ng/mL, PeproTech). After having been cultured for 10 to 14 days, the total number of spheres in every well was counted.

### Cell proliferation assay

Cell proliferation was assessed with cell counting kit-8 (CCK-8) assay (16). Cells were seeded into the 96-well plate (1 * 10^3^ cells per well). After having been cultured for 1, 2, 3, 4, and 5 days, the CCK-8 assay was performed as previously described to detect cell viability. The OD value is recorded at 490 nm.

### Cell viability assay and half-maximal drug inhibitory concentration (IC50)

Cell viability was detected with 3-(4,5-dimethylthiazol-2-yl)-2,5-diphenyltetrazolium bromide (MTT) assay as a previous study had described (17). Briefly, about 5,000 cells were seeded in the 96-well plate, and 48 h later MTT would be performed for the detection of cell viability. For the detection of IC50, cells were incubated overnight and treated with cisplatin at different concentrations (from 0.1 to 50 µm/L). After 72 h, cell viability was measured, and IC50 value was calculated with GraphPad Prism Software 6.0.

### Cell apoptosis assay

Cell apoptosis rate was investigated with Annexin V-APC assay and flow cytometry as previously described (18). Cells were washed with PBS and subsequently cultured for 30 min at 37°C after adding 5 µL Annexin V-phycoerythrin. Then, the cells were scanned, and the apoptosis rate was analyzed with flow cytometry.

### RNA extraction and real-time quantitative PCR

Total RNA was extracted with Trizol reagent following the product instruction. RNA reverse transcription and RT-qPCR were performed with Takara PrimeScript™ RT reagent Kit and Premix Ex Taq™ II (Takara Bio, Inc., Otsu, Japan) according to the manufacturer’s protocol. GAPDH was used as internal control, and RNA expression was assessed with the quantification approach (2^−ΔΔCt^ method). The primers used are as follows: PRKG1 forward: 5′-GCTGTAACCTGCCTTGTGATT-3′, reverse: 5′-GATGTGCTCCTGCTGTCTTGT-3′; GAPDH forward: 5′-TGACTTCAACAGCGACACCCA-3′, reverse: 5′-CACCCTGTTGCTGTAGCCAAA-3′.

### Statistical analysis

The difference of PRKG1 expression in cancer and normal tissues was analyzed with *t*-test. The associations between PRKG1 expression and clinicopathological characteristics were evaluated using chi-squared test or Mann–Whitney test. Cox proportional hazard regression model was adopted to conduct the estimate of prognosis. Survival analysis was performed with the Kaplan–Meier method. *P*-value <0.05 was considered to be statistically significant. All statistical analyses were conducted using SPSS 24.0 software package, R software 4.2.0, or GraphPad Prism Software 6.0. All of the experiments were performed in triplicate and repeated at least three times.

## Results

### PRKG1 is downregulated in bladder cancer tissues

**Figure 1:**
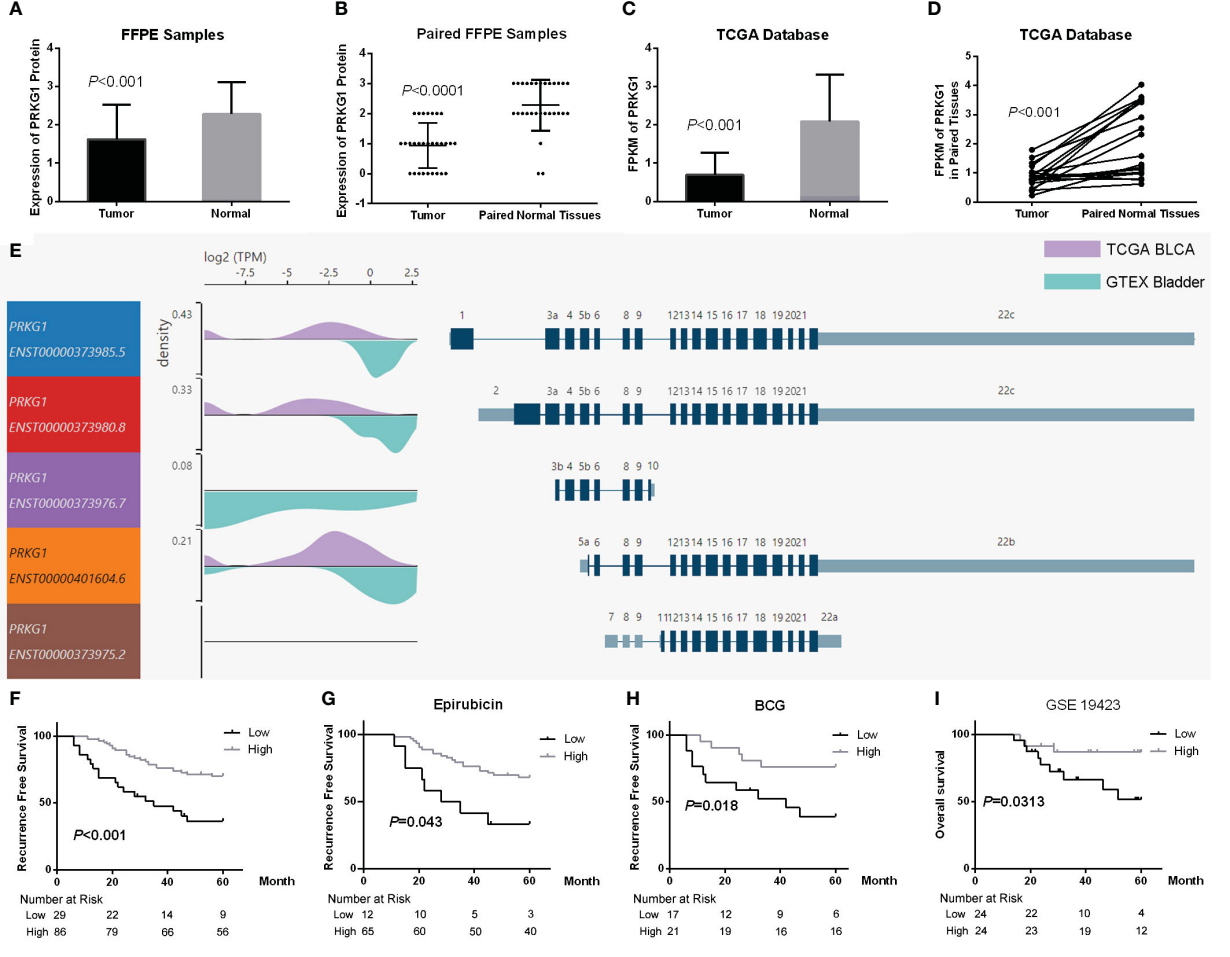
Expression of PRKG1 and the potential of prognosis. **(A)** Expression of PRKG1 protein in BCa and normal tissues. **(B)** Expression of PRKG1 protein in BCa and paired normal tissues. **(C)** FPKM of PRKG1 in TCGA BLCA samples (BCa and normal tissues). **(D)** FPKM of PRKG1 in BCa and paired normal tissues from TCGA BLCA database. **(E)** Expression of PRKG1 transcripts in BLCA samples and normal bladder samples from GTEx database. **(F)** RFS of patients received TURBT (lower PRKG1 related to poor prognosis), both in TURBT combined with intravesical epirubicin therapy **(G)** and intravesical BCG immunotherapy **(H)**. **(I)** Five-year OS of BCa patients who received TURBT combined with intravesical BCG immunotherapy (GSE19423).

The expression of PRKG1 is assessed in samples from TCGA database and FFPE samples. In FFPE samples, the expression score of PRKG1 protein in BCa tissues was significantly lower than in normal tissues (**Figure 1A**) and paired adjacent normal tissues (**Figure 1B**). ISH image and the related expression score are shown in **Supplementary Figure S1**. The results were also observed at RNA level in TCGA BLCA database (**Figures 1C, D**). The expression of PRKG1 transcripts is shown in **Figure 1E**, which was lower in BLCA samples than in normal bladder samples from GTEx database in general.

### Loss of PRKG1 predicts poor prognosis and response to BCG

To explore the clinical significance of PRKG1 expression in BCa, the correlation between PRKG1 protein expression and the clinical characteristics was analyzed. As shown in **Table 1**, a lower expression of PRKG1 was associated with higher tumor grade, T stage, and muscle invasion. In 115 patients who received TURBT, lower expression predicted worse recurrence-free survival (RFS) rate (**Figure 1F**), no matter if the patients received intravesical epirubicin therapy (**Figure 1G**) or BCG immunotherapy (**Figure 1H**). Cox regression analysis revealed that muscle invasion and lower PRKG1 were indicators of tumor recurrence (**Table 2**). In GSE19423 dataset, the Kaplan–Meier analysis indicated that a lower PRKG1 expression was related to poor 5-year overall survival (OS) in T1 patients who received intravesical BCG immunotherapy (**Figure 1I**). In addition, the role of low PRKG1 expression in predicting the recurrence rate of TURBT was analyzed with ROC curves. The results showed that a low PRKG1 expression might be used to predict the response to BCG (AUC: 1 year—0.79, 3 years—0.67, and 5 years—0.72; **Supplementary Figure S2A**), while it is not for epirubicin. Based on GSE19423 dataset, a low PRKG1 expression might also be used to predict the OS in patients who received BCG immunotherapy (AUC: 3 years—0.64 and 5 years—0.69; **Supplementary Figure S2B**).

**Table 2:** Cox regression analysis for tumor recurrence of patients with bladder cancer.

Parameter	HR (95% CI)	*P*
Age (<65 vs ≥65)	6.808 (1.872~24.763)	0.004
Gender (Male vs Female)	1.243 (0.465~3.327)	0.665
Grade (Low vs High)	1.010 (0.503~2.029)	0.978
Muscle Invasion (Yes vs No)	1.581 (1.085~2.305)	0.017
PRKG1 (2+/3+ vs 0/1+)	0.571 (0.398~0.821)	0.003

HR, Hazard ratio. 95% CI, 95% Confidence interval.

### PRKG1 inhibits BCa cell migration and invasion

**Figure 2:**
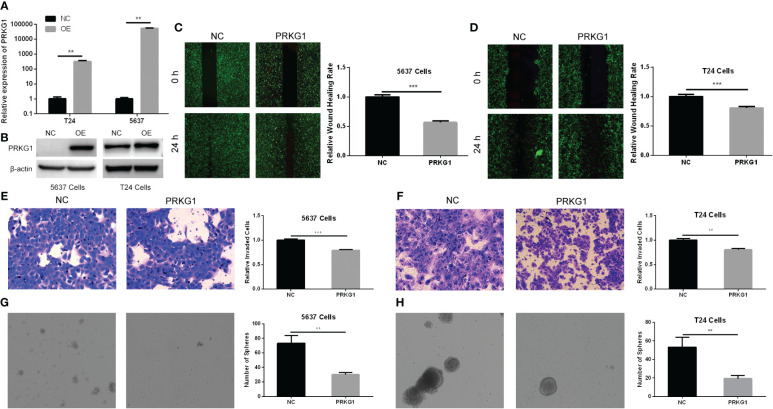
PRKG1 inhibits cell migrative, invasive, and spheroidizing ability. Validation of over-expression of PRKG1 mRNA **(A)** and protein **(B)**. In the monolayer wound healing assay, PRKG1 inhibits both 5637 **(C)** and T24 **(D)** cell migration. In Transwell invasion assay, PRKG1 inhibits both 5637 **(E)** and T24 **(F)** cell invasion. In sphere formation assay, PRKG1 inhibits spheroid formation of 5637 **(G)** and T24 **(H)** cells. **P < 0.01; ***P < 0.001.

To explore the role of PRKG1 in cellular migration and invasion, we detected cell migration ability with a monolayer wound healing assay and the invasion ability with Transwell invasion assay. After having been transfected with PRKG1 lentivirus or control, the expression of PRKG1 mRNA and protein was detected to verify the over-expression (**Figures 2A, B**). As shown in **Figures 2C, D**, the over-expression of PRKG1 inhibited both 5637 and T24 cell migration significantly, which can also be observed in the cell Transwell invasion assay (**Figures 2E, F**). However, the degree of suppression was not very high, although the difference was significant.

### PRKG1 inhibits the spheroidizing ability of BCa cells

The stem-cell-like properties of BCa cells were verified with tumor sphere formation assay. In cells transfected with PRKG1 lentivirus, the spheroids were less than the NC group (**Figures 2G, H**). Thus, the results indicated that the cells over-expressing PRKG1 had lower stemness and ability to form spheroids.

### Over-expression of PRKG1 suppresses cell proliferation and cell viability

**Figure 3:**
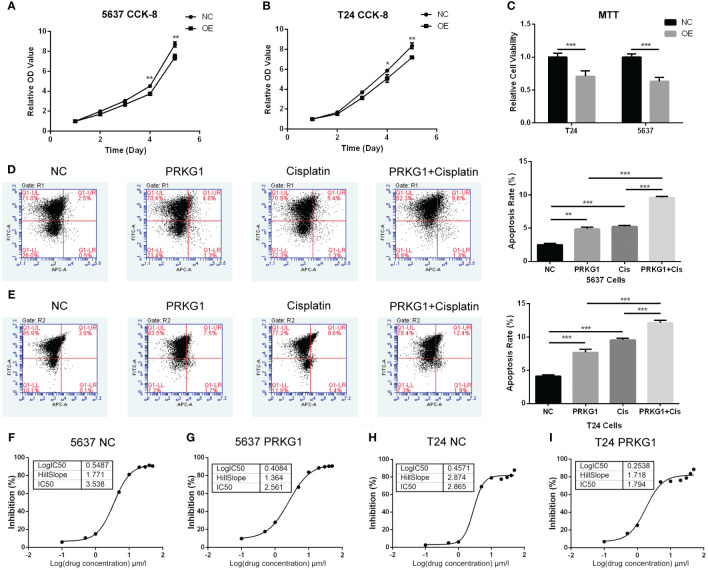
PRKG1 inhibits cell proliferation and viability, induces cell apoptosis, and enhances chemosensitivity of cisplatin. The results of CCK-8 indicates the inhibition of cell proliferation [**(A)** 5637 cells and **(B)** T24 cells] of PRKG1 over-expression. **(C)** Over-expression of PRKG1 inhibits cell viability. PRKG1 induces 5637 **(D)** and T24 **(E)** cell apoptosis and enhances apoptosis rate when treated with cisplatin. **(F, G)** IC50 of 5637 NC cells and PRKG1 over-expression cells. Over-expression of PRKG1 reduces the value of IC50. **(H, I)** IC50 of T24 NC cells and PRKG1 over-expression cells. **P < 0.01; ***P < 0.001.

CCK-8 and MTT assay were performed to assess cell proliferation and viability. For the cell proliferation assay, PRKG1 inhibited the cell proliferation rate at the 4th and 5th day in 5637 (**Figure 3A**) and T24 (**Figure 3B**) cells. In cells that over-expressed PRKG1, the viability is significantly lower than the NC group (**Figure 3C**). The results showed that PRKG1 could suppress BCa cell proliferation and viability.

### PRKG1 induces chemosensitivity to cisplatin

To probe the role of PRKG1 in chemoresistance, we evaluated the apoptosis rate and cisplatin IC50 of BCa cells with over-expressed PRKG1. Over-expression of PRKG1 induced cell apoptosis in T24 and 5637 cells, and the apoptosis rate was furthermore increased when treated with cisplatin (**Figures 3D, E**). A higher IC50 value was considered to be correlated with clinical chemoresistance to drugs. IC50 in 5637 cells with over-expressed PRKG1 was 2.561 µmol/L, while IC50 in 5637 NC cells was 3.538 µmol/L (**Figures 3F, G**). Similar results could be observed in T24 cells (**Figures 3H, I**). The over-expression of PRKG1 could increase the apoptosis rate when treated with cisplatin and reduce the IC50 value, which meant that PRKG1 promoted chemosensitivity.

### Relationship between the expression of PRKG1 and TME and tumor heterogeneity

**Figure 4:**
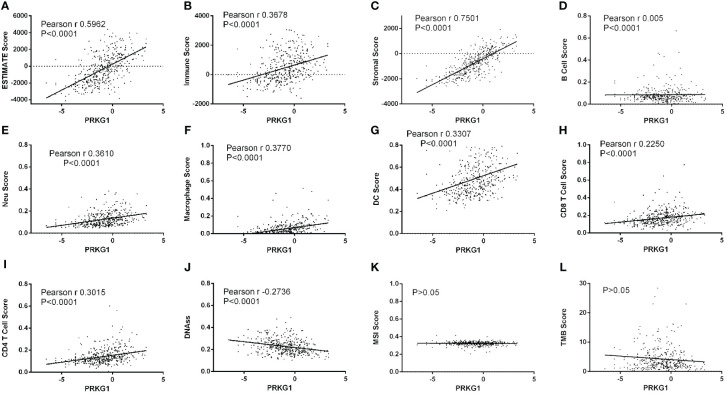
Relationship between the expression of PRKG1 and TME and tumor heterogeneity. The expression of PRKG1 is positively related to ESTIMATE score **(A)**, immune score **(B)**, and stromal score **(C)**. In the analysis of cell infiltration, PRKG1 is positively correlated with B cell **(D)**, neutrophil **(E)**, macrophage **(F)**, DC **(G)**, CD8+ T cell **(H)**, and CD4+T cell **(I)** infiltration. In the analysis of tumor heterogeneity, PRKG1 is negatively correlated with DNAss **(J)** and not correlated with MSI **(K)** or TMB score **(L)**.

To explore the roles of PRKG1 in BCa TME and tumor heterogeneity, the relationship between PRKG1 and TME index and tumor heterogeneity in TCGA BLCA samples was analyzed. It was found that the expression of PRKG1 positively related to ESTIMATE score (**Figure 4A**), immune score (**Figure 4B**), and stromal score (**Figure 4C**). The extent of immune cell infiltration was also analyzed, which showed that PRKG1 was positively correlated with B cell, CD4^+^T cell, CD8^+^ T cell, neutrophil, macrophage, and dendritic cell (DC) infiltration (**Figures 4D–I**). In the analysis of tumor heterogeneity, PRKG1 was negatively correlated with DNAss (**Figure 4J**), and no correlation was found with MSI and TMB score (**Figures 4K, L**). Based on the PRKG1 expression in the TCGA–BLCA cohort, the mutant landscape was organized. Mutations were more frequently observed in low-expressed PRKG1 group, especially missense mutations (**Supplementary Figure S3A**). In the TCGA–BLCA cohort, KDM6A, FGFR3, STAG2, OBSCN, BIRC, ADGRV1, DST, ABCA13, USP34, and TENM3 had more frequent mutation in samples with low PRKG1 expression.

### Pathway enrichment of PRKG1 co-expressed genes

PRKG1-co-expressed genes in TCGA–BLCA cohort were analyzed. The top 50 positively correlated genes and 50 negatively correlated genes were generated, and pathway enrichment was performed based on Kyoto Encyclopedia of Genes and Genomes (KEGG) and Gene Ontology (GO) database. In the KEGG analysis, the genes were enriched in focal adhesion, PI3K-Akt signaling, ECM–receptor interaction, etc. (**Supplementary Figure S3B**). As for GO biological process, the terms included biological adhesion, regulation of cell differentiation, tube morphogenesis, etc. (**Supplementary Figure S3C**). Similar to a previous study which showed that PRKG1 inhibited the activity of MAPK/ERK pathway in the epithelial ovarian pathway (12), we also detected the expression of MEK1/2 and p-MEK1/2 in 5637 and T24 cells. The results showed that the expression of both MEK1/2 and p-MEK1/2 did not change significantly (**Supplementary Figure S4**), indicating a different mechanism of PRKG1 in bladder cancer development.

### Relationship between the expression of PRKG1 and immune checkpoint-related genes

**Figure 5:**
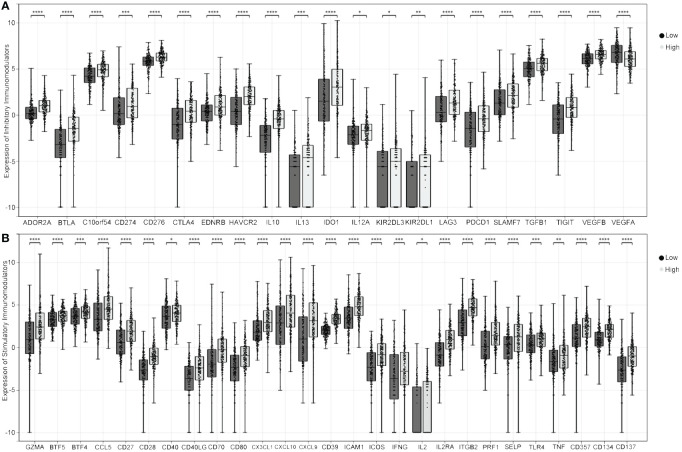
Relationship between PRKG1 and immune checkpoint-related genes. In samples with high PRKG1 expression, the expression of most inhibitory **(A)** and stimulatory genes **(B)** is higher than in low PRKG1 expression. *P < 0.05; **P < 0.01; ***P < 0.001; ****P < 0.0001.

The expression of 60 immune checkpoint-related inhibitory and stimulatory genes in a previous study (19) was downloaded from UCSC. Most inhibitory (**Figure 5A**) and stimulatory genes (**Figure 5B**) in samples with high PRKG1 expression are higher than the low-PRKG1 group, which represented a different TME in different PRKG1 expression samples.

## Discussion

PRKG1 was identified as one of the protein kinases that belonged to the serine/threonine family (20). According to previous studies, PRKG1 can be activated when the biological signaling of cGMP was mediated by nitric oxide (NO) (21). After binding to soluble guanylyl cyclase (GC), also known as the prosthetic heme group, NO at nanomolar levels can cause activation of the enzyme (22). The elevation of cGMP was caused by NO–GC, which conversed GTP to cGMP, resulting in the initiation of the cGMP signaling pathway and subsequent physiological changes (23). It is well known that the activation of the cGMP–PRKG1 axis regulates calcium homeostasis, smooth muscle contraction, platelet activation and adhesion, cardiac function, and other processes (24). Current medicine, such as PDE5 inhibitors, can successfully target the NO–GC–cGMP signaling for treatment of a lot of vascular diseases such as pulmonary hypertension, angina pectoris, and erectile dysfunction (25). Interestingly, based on the discovery of cGMP–PRKG1–MAPK signaling in melanoma cells, a prospective cohort study carried out by Li et al. suggested that the use of sildenafil, one of the PDE5 inhibitors, may be correlated with an increased risk of developing melanoma (26). Another study also found that the cGMP–PRKG1 pathway was the link between sildenafil usage and increased melanoma risk (27). However, suppression of PDE5 with antisense transcripts or PDE5 inhibitors promotes colon cancer cell apoptosis and inhibits growth, which is involving the activation of the cGMP/PRKG1 signaling pathway (28). Well then, what is the role of PRKG1 in cancer development?

A previous study has revealed that PRKG1 is downregulated in tumors from liver, pancreas, lung, and colon and can regulate cancer cellular function (11). In breast cancer cells, PRKG1 promotes cell apoptosis via hyperactivating DAPK2 with phosphorylating it at Ser^299^ (29). Our study explored the role of PRKG1 in BCa; the results showed that PRKG1 inhibited cell proliferation, migration, invasion, and stemness and induced cell apoptosis. When treated with cisplatin, the over-expression of PRKG1 reduced the IC50 value, which meant increased chemosensitivity. DNAss is the stemness index derived based on methylation data. The results showed that PRKG1 expression was negatively correlated with DNAss, which indicated lower PRKG1 corresponded to stronger tumor cell stemness. Thus, activation or over-expression of PRKG1 might play an anti-tumor role in bladder cancer.

A recent research divided MIBC into three groups according to the transcriptomic data—one group with a high expression of collagen-related genes, PDGFRB, and PRKG1 had the poorest overall survival (30). In the present study, we found that the expression of PRKG1 mRNA and protein was lower in BCa tissues than in normal bladder tissues. The lower expression of PRKG1 is associated with a higher tumor grade and muscle invasion. The loss of PRKG1 in BCa tissues was associated with a poor overall survival rate and response to TURBT combined with intravesical instillations. What is more, low PRKG1 expression predicted poor response and overall survival when treated with BCG. As a common immunotherapy of BCa, non-response to BCG meant abnormal tumor immune microenvironment.

In the TME treated with BCG, BCG infiltrates the urothelium and is taken up by macrophages, initiating local immune activation (31, 32). The dominant pathway for the early activation of macrophages involves pro-inflammatory cytokines. DCs are responsible for the induction of antigen-specific immunity and promote the killing of cancer cells by NKT cells and γ/δT cells (32, 33). In addition to innate immunity, adaptive immunity, especially T cells, is critical to the effectiveness of BCG therapy. T cells, including CD4^+^ and CD8^+^ T cells, are found in the urine and bladder mucosa of BCG-treated patients (34). A previous study showed that injection of T cells from BCG-cured mice to tumor-bearing mice resulted in tumor rejection, indicating the presence of tumor-specific memory T cells (33). The absence of T cells in an animal model caused loss of BCG effectiveness (33). In general, the effectiveness of BCG treatment depends on the extent and persistence of BCG-induced immune responses (35). In the analysis of infiltration level, the expression of PRKG1 is positively associated with the stromal and immune scores and the infiltrated level of tumor-related immune cells, including T cells, macrophages, DCs, etc. The results suggested that PRKG1 participated in tumor immunotherapy by regulating immune cell infiltration, and patients with higher PRKG1 showed higher enhanced innate immunity and adaptive immunity to bladder tumors. In addition to immune cells, chemokines and cytokine are also important in TME. The immunomodulators are the medium of infiltrating immune cells and tumor cells, which can recruit immune cells into the TME, mediate immune cells to kill tumor cells, or protect tumor cells. Some chemokines play important roles in recruiting immune cells, such as CXCL9 and CXCL10, which can recruit NK cells, NKT cells, and macrophages to the TME and increases the cytotoxic activity against tumor cells (36). In the present study, the expression of most stimulatory and inhibitory immunomodulators was significantly lower in the low-PRKG1-expressed group, which might be attributed to the downregulation of pre-existing immune cells infiltrating, further indicating the regulatory role of PRKG1 in tumor immunotherapy.

## Conclusion

Generally, a lower expression of PRKG1 could be observed in bladder cancer tissues compared with normal tissues, which is also related to higher tumor grade and muscle invasion. What is more, loss of PRKG1 predicts poor prognosis (RFS and OS) in patients treated with BCG. The expression of PRKG1 is positively correlated with immunomodulators and immune cell infiltration. The results indicate the regulatory role of PRKG1 in BCa immunotherapy and its potential to be used as a biomarker for tumor immunotherapy.

## Data availability statement

The original contributions presented in the study are included in the article/**Supplementary Material**. Further inquiries can be directed to the corresponding authors.

## Ethics statement

The studies involving humans were approved by Ethics Committee of the Second Affiliated Hospital of Soochow University. The studies were conducted in accordance with the local legislation and institutional requirements. The participants provided their written informed consent to participate in this study.

## Author contributions

LJ: Funding acquisition, Investigation, Methodology, Writing – original draft. TC: Data curation, Investigation, Software, Writing – review & editing. HS: Formal analysis, Investigation, Visualization, Writing – review & editing. GD: Investigation, Methodology, Writing – review & editing. QY: Formal analysis, Software, Writing – review & editing. FY: Methodology, Visualization, Writing – review & editing. XL: Resources, Validation, Writing – review & editing. BX: Conceptualization, Funding acquisition, Project administration, Resources, Validation, Writing – review & editing.
