# Supplementary material for: The clinical significance and anti-tumor role of PRKG1 in bladder cancer
Source: Front Immunol. 2024 Jul 30;15:1442555. doi: 10.3389/fimmu.2024.1442555 (PMC11319154; doi:10.3389/fimmu.2024.1442555)
Supplement: Supplementary file 1 [file Image_1.pdf]

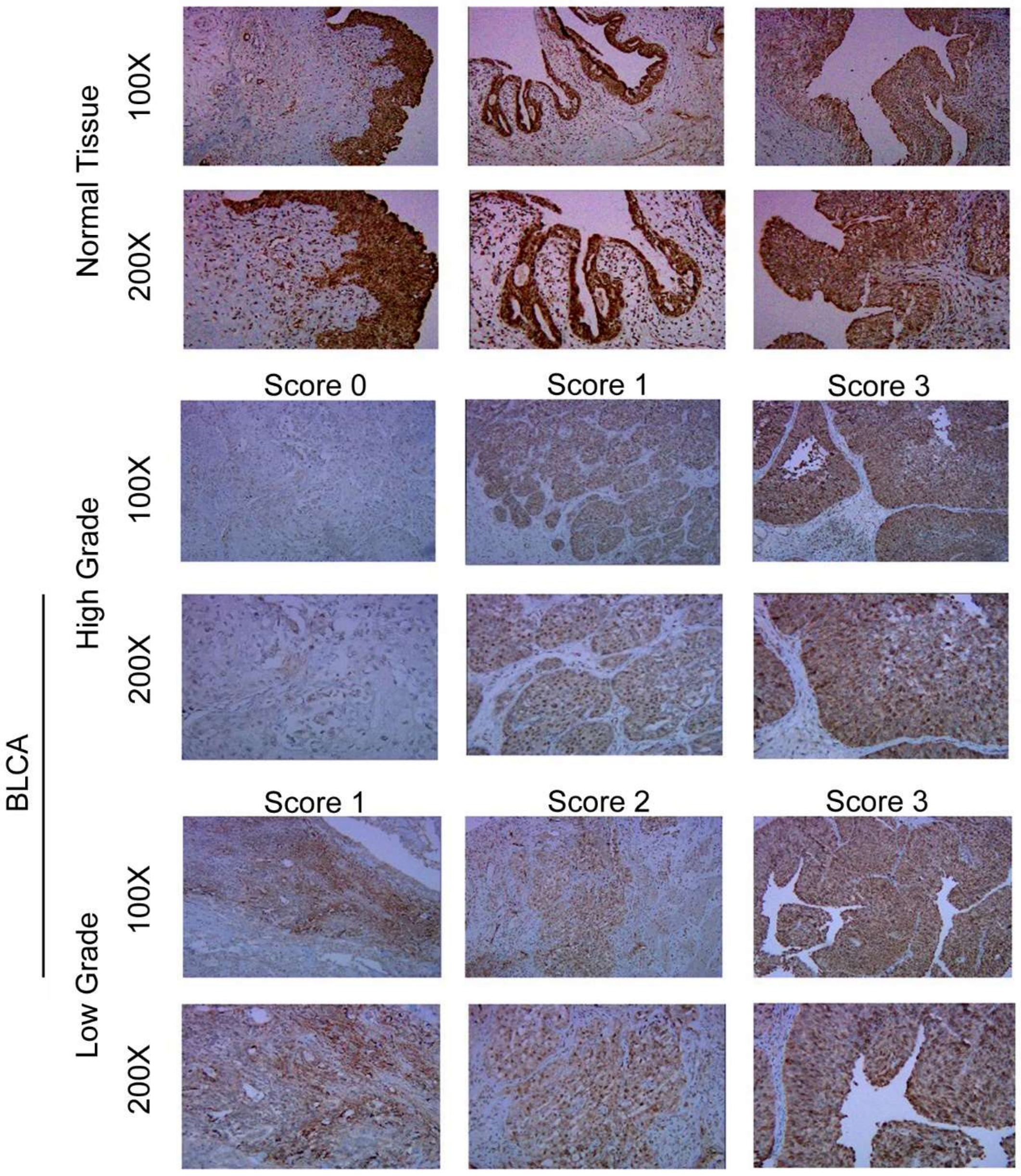

Supplementary Figure 1. Expression score of PRKG1 protein in BCa tissues and normal bladder tissues.
