# Supplementary material for: The clinical significance and anti-tumor role of PRKG1 in bladder cancer
Source: Front Immunol. 2024 Jul 30;15:1442555. doi: 10.3389/fimmu.2024.1442555 (PMC11319154; doi:10.3389/fimmu.2024.1442555)
Supplement: Supplementary file 2 [file Image_2.pdf]

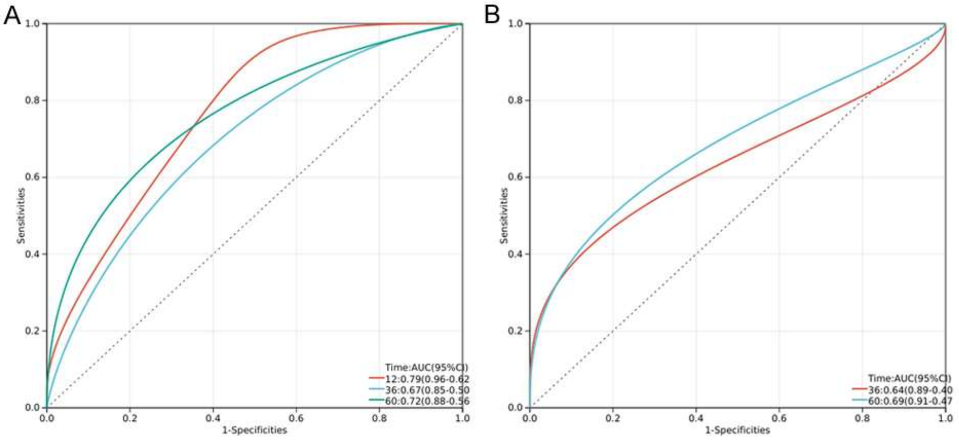

Supplementary Figure 2. ROC curve of patients received TURBT combined with intravesical BCG immunotherapy. A. ROC curve for RFS in FFPE samples. B. ROC curve for OS in sample from GSE19423 dataset. (Time: month; AUC: area under curve.)
