# Supplementary material for: The clinical significance and anti-tumor role of PRKG1 in bladder cancer
Source: Front Immunol. 2024 Jul 30;15:1442555. doi: 10.3389/fimmu.2024.1442555 (PMC11319154; doi:10.3389/fimmu.2024.1442555)
Supplement: Supplementary file 3 [file Image_3.pdf]

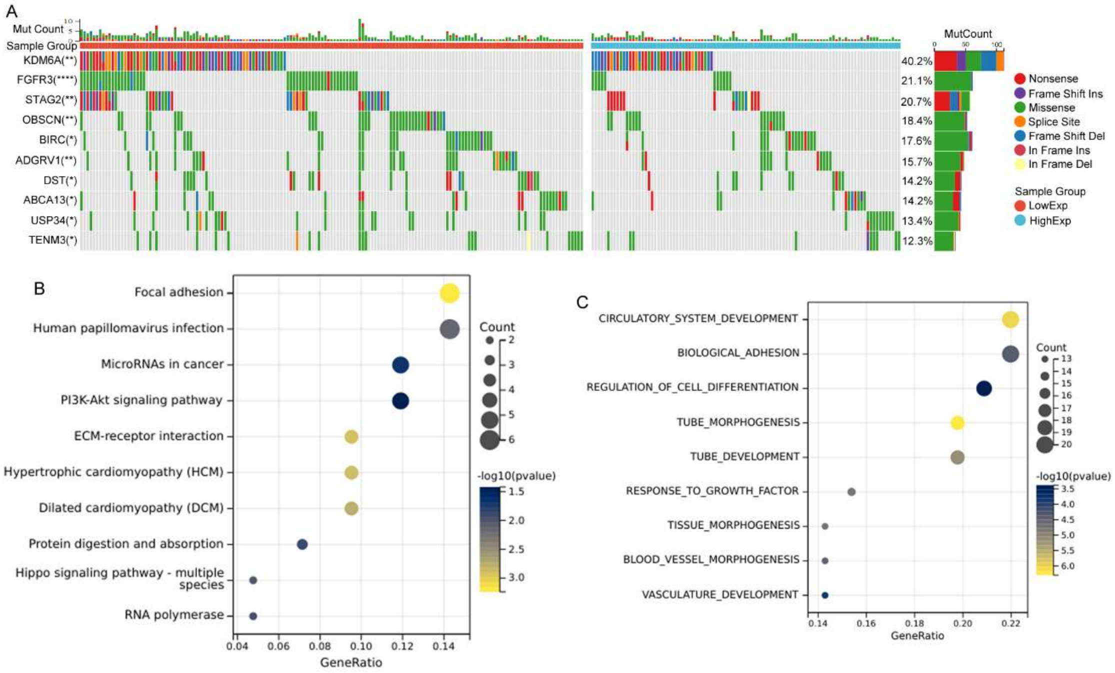


**Supplementary Figure 3.** A.Mutant landscape of samples from TCGA-BLCA cohort. Mutations are more frequently observed in low-expressed PRKG1 group. KDM6A, FGFR3, STAG2, OBSCN, BIRC, ADGRV1, DST, ABCA13, USP34 and TENM3 have more frequent mutation in samples with low PRKG1 expression. B. KEGG pathway enrichment of PRKG1 co-expressed genes. C. Go cellular process enrichment of PRKG1 co-expressed genes.
