# Supplementary material for: The clinical significance and anti-tumor role of PRKG1 in bladder cancer
Source: Front Immunol. 2024 Jul 30;15:1442555. doi: 10.3389/fimmu.2024.1442555 (PMC11319154; doi:10.3389/fimmu.2024.1442555)
Supplement: Supplementary file 4 [file Image_4.pdf]

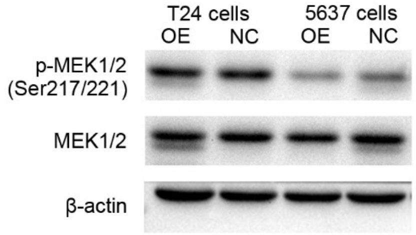

Supplementary Figure 4. Expression of MEK1/2 and p-MEK1/2 (Ser217/221) in T24 and 5637 cells. OE: PRKG1 overexpressed group; NC: Negative control.
